# Supplementary material for: The evolving genetic landscape of telomere biology disorder dyskeratosis congenita
Source: EMBO Mol Med. 2024 Aug 28;16(10):16. doi: 10.1038/s44321-024-00118-x (PMC11473520; doi:10.1038/s44321-024-00118-x)
Supplement: Supplementary file 1 — Table EV1 [file 44321_2024_118_MOESM1_ESM.docx]

**Table EV1: Small nuclear and C/D box RNA’s fold change**

Table listing differentially regulated (log2fold) *snRNA* and *snoRNA* genes observed in *ZCCHC8* patients Vs controls.
