# Supplementary material for: The evolving genetic landscape of telomere biology disorder dyskeratosis congenita
Source: EMBO Mol Med. 2024 Aug 28;16(10):16. doi: 10.1038/s44321-024-00118-x (PMC11473520; doi:10.1038/s44321-024-00118-x)
Supplement: Supplementary file 2 — Appendix [file 44321_2024_118_MOESM2_ESM.pdf]

Appendix

Table of Contents

Appendix Tables

Table S1: --2
Table S2: --2
Table S3: --2
Table S4: --3
Table S5: --4
Table S6: --5
Appendix references--6-10

Appendix Table S1: Targeted bone marrow failure gene panel

ACD	DDX41	FANCE	JAGN1	POLA1	RPL5	SBDS
ADA2	DKC1	FANCF	KIF23	POT1	RPL9	SEC23B
ANKRD26	DNAJC21	FANCG	KLF1	RAD51	RPS10	SHQ1
BRCA1	DNAJC3	FANCI	LIG4	RAD51C	RPS17	SLX4
BRCA2	DUT	FANCL	LPIN2	RBM8A	RPS19	SP1
BRIP1	EFL1	FANCM	MAD2L2	RECQL4	RPS24	SRP54
C15orf41	ELANE	FYB1	MECOM	RFWD3	RPS26	SRP72
CDAN1	ERBB3	G6PC	MPL	RMRP	RPS27	STN1
CEBPA	ERCC4	G6PC3	MYSM1	RPL11	RPS28	TAZ
CSF3R	ERCC6L2	GATA1	NAF1	RPL15	RPS29	TERC
CTC1	ETV6	GATA2	NHP2	RPL18	RPS7	TERT
CXCR4	FANCA	GFI1	NOP10	RPL26	RTEL1	THPO
CYCS	FANCB	GRHL2	NPM1	RPL27	RUNX1	TINF2
DCLRE1B	FANCC	HAX1	PALB2	RPL31	SAMD9	TP53
FANCD2	HOXA11	PARN	RPL35A	SAMD9L	TYMS	UBE2T
USB1	VPS45	WAS	WRAP53	XRCC2	ZCCHC8	

Appendix Table S2: Sample filtering

Criteria	samples	families
Total number	404	329
Males, excluding those in AD or AR families	157	146
Males, excluding those in AD or AR families, uncharacterised	131	120

AD = autosomal dominant; AR = autosomal recessive

Appendix Table S3. Variant filtering

Criteria	variants	genes	samples	families
X-linked, somewhat rare (MAF<0.05)	3145	687	.	.
X-linked, pass filter, not reported on gnomAD or Kaviar databases (novel)	389	282	240	216
Novel X-linked variants, homozygous or hemizygous	175	150	127	112
Novel X-linked variants in males, excluding those in AD or AR families	128	109	81	76
Novel X-linked variants in males, excluding those in AD or AR families, in **uncharacterised families**	110	94	71	66
Novel X-linked variants in males, excluding those in AD or AR families, in genes seen in **two or more uncharacterised families**	21	11	23	19
Novel X-linked variants in males, excluding those in AD or AR families, in genes seen **segregating in two or more uncharacterised multiplex families**	2	1	4	2

Appendix Table S4: Genes in which novel X-linked variants were identified in two or more unrelated individuals

Patient UPN	Inheritance*	Gene	DNA variant	AA Change
DC_2479	S	BMX	c.243G>A	p.Gln81=†
DC_3636	S	BMX	c.1370T>C	p.Phe457Ser
DC_2324; DC_2422	XL	POLA1	c.1846A>G	p.Arg616Gly
DC_1361; DC_1362	2MS	POLA1	c.3437A>G	p.Tyr1146Cys
DC_4627	S	POLA1	c.1609C>A	p.Leu537Ile
DC_2887	S	CFAP47	c.344C>T	p.Thr115Met
DC_1663; DC_1666	2MS	CFAP47	c.1847T>C	p.Ile616Thr
DC_4490	S	PHF8	c.2110_2127dup	p.Tyr704_Asp709dup
DC_8516**	2MS	PHF8	c.1250-10_1250-4del	N/A
DC_3551	S	PFKFB1	c.581T>C	p.Leu194Pro
DC_862	S	PFKFB1	c.157C>T	p.Arg53Ter
DC_4047	S	RBMXL3	c.1033T>C	p.Ser345Pro
DC_4454	S	RBMXL3	c.1193_1194insGGCCGCTCGCCCGG	p.Asp398GlufsTer114
DC_594	S	XPNPEP2	c.349T>C	p.Tyr117His
DC_3899**	2MS	XPNPEP2	c.1886T>C	p.Leu629Pro
DC_2887	S	GPR112	c.11A>G	p.His4Arg
DC_4047	S	GPR112	c.4510A>G	p.Met1504Val
DC_3049	S	MAGEC3	c.1777T>G	p.Ser593Ala
DC_1439	S	MAGEC3	c.1846C>G	p.Gln616Glu
DC_2292; DC_2816	S:S	NUP62CL	c.344T>C	p.Met115Thr
DC_2101	S	F8	c.4652C>T	p.Ala1551Val
DC_2695	S	F8	c.2765C>T	p.Ser922Phe

* Pattern of inheritance: S = simplex case; 2MS = two male sibs; XL = X-linked; ** affected sib unavailable; † = splice region

Appendix Table S5: Primers used in these studies

Primer name	Primer sequence	Chemistry	Mastermix
hL1-ORF1-F	TCAAAGGAAAGCCCATCAGACTA	Sybr green	PowerUp Sybrgreen
hL1-ORF1-R	TTGGCCCCCACTCTCTTCT	Sybr green	PowerUp Sybrgreen
hL1-ORF2-F	GAGAGGATGCGGAGAAATAGGA	Sybr green	PowerUp Sybrgreen
hL1-ORF2-R	GGATGGCTGGGTCAAATGGT	Sybr green	PowerUp Sybrgreen
hTR 3'_Fw	AGTTCGCTTTCCTGTTGGTG	Sybr green	PowerUp Sybrgreen
hTR 3'_Rv	AGGTTTGGGGGTTCACAAG	Sybr green	PowerUp Sybrgreen
GAPDH_Fw	TGCACCACCAACTGCTTAGC	Sybr green	PowerUp Sybrgreen
GAPDH_Rv	GGCATGGACTGTGGTCATGAG	Sybr green	PowerUp Sybrgreen
10q_Fw	AAAGCGGGAAACGAAAAGC	Sybr green	PowerUp Sybrgreen
10q_Rv	GCCTTGCCTTGGGAGAATCT	Sybr green	PowerUp Sybrgreen
15q_Fw	GCGTGGCTTTGGGACAACT	Sybr green	PowerUp Sybrgreen
15q_Rv	TGCAACCGGGAAAGATTTTATT	Sybr green	PowerUp Sybrgreen
9p_Fw	GAGATTCTCCCAAGGCAAGG	Sybr green	PowerUp Sybrgreen
9p_Rv	ACATGAGGAATGTGGGTGTTAT	Sybr green	PowerUp Sybrgreen
TaqMan probes			
Gene	Code	TaqMan	TaqMan Fast Advanced
TFRC	Hs00951083_m1	TaqMan	TaqMan Fast Advanced
PARN	Hs003777733_m1	TaqMan	TaqMan Fast Advanced
ZCCHC8	Hs00214116_m1	TaqMan	TaqMan Fast Advanced
GAPDH	Hs0275899_g1	TaqMan	TaqMan Fast Advanced
GAS5	Hs05021116_g1	Taqman	TaqMan Fast Advanced
NORAD	Hs05023184_s1	Taqman	TaqMan Fast Advanced
XCIP assay			
Humara Fwd	[FAM]-AGC GTG CGC GAA GTG ATC CAG		
Humara Rev	GCTGTGAAGGTTGCTGTTCCTCAT		

Appendix Table S6: Antibodies used in the study

Antibody	Dilution
**POT1**	1:2000 WB
**TPP1**	1:2000 WB
**Phospho ATR**	1:2500 WB
**Phospho CHK1**	1:2500 WB
**Phospho P53**	1:1000 WB
**RPA70**	1:1000 WB<br>1:200 PLA
**α-Tubulin**	1:1000 WB
**GFP**	1:1000 WB
**PRIM2A**	1:1000 WB
**PRIM1**	1:2500 WB
**Histidine**	1:2500 WB
**FLAG tag**	1:2500 WB
**HA-tag**	1:2500 WB
**ZCCHC8**	1:1000 WB
**MYC-tag**	1:2500 WB
**53BP1**	1:200 PLA
**TRF2**	1:100 PLA

Appendix References

- Alder et al. Short telomeres are a risk factor for idiopathic pulmonary fibrosis. Proc Natl Acad Sci U S A. 2008 Sep 2;105(35):13051-6.
- Anderson *et al.* Mutations in *CTC1*, encoding conserved telomere maintenance component 1, cause Coats plus. *Nat Genet* **44**, 338–342 (2012).
- Arun et al. Spectrum of *ELANE* mutations in congenital neutropenia: a single-centre study in patients of Indian origin. J Clin Pathol. 2018 Dec;71(12):1046-1050.
- Ballew et al. Germline mutations of regulator of telomere elongation helicase 1, RTEL1, in Dyskeratosis congenita. Hum Genet. 132(4):473-80 (2013).
- Baralle et al. Splicing in action: assessing disease causing sequence changes. J Med Genet. 2005 Oct;42(10):737-48.
- Borie et al. Regulator of telomere length 1 (*RTEL1*) mutations are associated with heterogeneous pulmonary and extra-pulmonary phenotypes. Eur Respir J. 2019 53(2):1800508 (2019).
- Calado et al. Constitutional telomerase mutations are genetic risk factors for cirrhosis. Hepatology. 2011 May;53(5):1600-7.
- Cardoso et al. Germline heterozygous DDX41 variants in a subset of familial myelodysplasia and acute myeloid leukemia. Leukemia.30(10):2083-2086 (2016).
- Cardoso et al. Myelodysplasia and liver disease extend the spectrum of RTEL1 related telomeropathies. Haematologica. 102(8):e293-e296 (2017).
- Carrillo et al. High resolution melting analysis for the identification of novel mutations in DKC1 and TERT genes in patients with dyskeratosis congenita. Blood Cells Mol Dis. 2012 Oct 15-Dec 15;49(3-4):140-6.
- Chan et al. Spectrum of Germline Mutations Within Fanconi Anemia–Associated Genes Across Populations of Varying Ancestry, *JNCI Cancer Spectrum*, Volume 5, Issue 1, February 2021, pkaa117.
- Clericuzio et al. Clericuzio-type poikiloderma with neutropenia syndrome in three sibs with mutations in the C16orf57 gene: delineation of the phenotype. Am J Med Genet A. 2010 Oct;152A(10):2588-94.
- Cmejla et al. Identification of mutations in the ribosomal protein L5 (RPL5) and ribosomal protein L11 (RPL11) genes in Czech patients with Diamond-Blackfan anemia. Hum Mutat. 2009 Mar;30(3):321-7.
- Collopy et al. Targeted resequencing of 52 bone marrow failure genes in patients with aplastic anemia reveals an increased frequency of novel variants of unknown significance only in SLX4. Haematologica. 2014 Jul;99(7):e109-11.
- Collopy et al. Triallelic and epigenetic-like inheritance in human disorders of telomerase. Blood. (2015) 126(2):176-84.
- Collopy et al. Targeted resequencing of 52 bone marrow failure genes in patients with aplastic anemia reveals an increased frequency of novel variants of unknown significance only in SLX4. Haematologica. 2014 Jul;99(7):e109-11.
- Cossu et al. A novel DKC1 mutation, severe combined immunodeficiency (T+B-NK-SCID) and bone marrow transplantation in an infant with Hoyeraal-Hreidarsson syndrome. Br J Haematol. 2002 Dec;119(3):765-8.
- Dai et al. Telomerase gene mutations and telomere length shortening in patients with idiopathic pulmonary fibrosis in a Chinese population. Respirology. 2015 Jan;20(1):122-8.

- Diaz de Leon et al. Telomere lengths, pulmonary fibrosis and telomerase (TERT) mutations. PLoS One. 2010 May 19;5(5):e10680.
- Dodson et al. From incomplete penetrance with normal telomere length to severe disease and telomere shortening in a family with monoallelic and biallelic PARN pathogenic variants. Hum Mutat. 2019 Dec;40(12):2414-2429.
- Du et al. Complex inheritance pattern of dyskeratosis congenita in two families with 2 different mutations in the telomerase reverse transcriptase gene. Blood. 2008 Feb 1;111(3):1128-30.
- Du et al. TERC and TERT gene mutations in patients with bone marrow failure and the significance of telomere length measurements. Blood. 2009 Jan 8;113(2):309-16.
- Duployez et al. Prognostic impact of DDX41 germline mutations in intensively treated acute myeloid leukemia patients: an ALFA-FILO study. Blood. 2022 Aug 18;140(7):756-768.
- Gutierrez-Rodrigues et al. Pathogenic TERT promoter variants in telomere diseases. Genet Med. 2019 Jul;21(7):1594-1602.
- Guo et al. Inherited bone marrow failure associated with germline mutation of ACD, the gene encoding telomere protein TPP1. Blood. 2014 Oct 30;124(18):2767-74.
- Heiss et al. X-linked dyskeratosis congenita is caused by mutations in a highly conserved gene with putative nucleolar functions. Nat Genet. 1998 May;19(1):32-8.
- Holme et al. Marked genetic heterogeneity in familial myelodysplasia/acute myeloid leukaemia. Br J Haematol. 2012 Jul;158(2):242-248.
- Homan et al. GATA2 deficiency syndrome: A decade of discovery. Hum Mutat. 2021 Nov;42(11):1399-1421.
- Justet et al. Safety and efficacy of pirfenidone and nintedanib in patients with idiopathic pulmonary fibrosis and carrying a telomere-related gene mutation. Eur Respir J. 2021 Feb 11;57(2):2003198.
- Kanegane et al. Identification of DKC1 gene mutations in Japanese patients with X-linked dyskeratosis congenita. Br J Haematol. 2005 May;129(3):432-4.
- Keller et al. CTC1 Mutations in a patient with dyskeratosis congenita. Pediatr Blood Cancer. 2012 59(2):311-4.
- Kirwan et al. Defining the pathogenic role of telomerase mutations in myelodysplastic syndrome and acute myeloid leukemia. Hum Mutat. 2009 30(11):1567-73.
- Kirwan et al. Exome sequencing identifies autosomal-dominant SRP72 mutations associated with familial aplasia and myelodysplasia. Am J Hum Genet. 2012 90(5):888-92.
- Knight et al. X-linked dyskeratosis congenita is predominantly caused by missense mutations in the DKC1 gene. Am J Hum Genet. 1999 65(1):50-8.
- Knight et al. Identification of novel DKC1 mutations in patients with dyskeratosis congenita: implications for pathophysiology and diagnosis. Hum Genet. 2001 108(4):299-303.
- Liu et al. De novo intronic GATA1 mutation leads to diamond-blackfan anemia like disease. Front Genet. 2023 10;14:1068923.
- Marrone et al. Functional characterization of novel telomerase RNA (TERC) mutations in patients with diverse clinical and pathological presentations. Haematologica. 2007 92(8):1013-20.

- Marx et al. A gain-of-function variant in the Wiskott-Aldrich syndrome gene is associated with a MYH9-related disease-like syndrome. *Blood Adv* (2022) 6 (18): 5279–5284.
- Minelli et al. The isochromosome i(7)(q10) carrying c.258+2t>c mutation of the SBDS gene does not promote development of myeloid malignancies in patients with Shwachman syndrome. Leukemia. 2009 23(4):708-11.
- Morgan et al. A common Fanconi anemia mutation in black populations of sub-Saharan Africa. Blood. 2005 105(9):3542-4.
- Mutsaers et al. Highly variable clinical manifestations in a large family with a novel GATA2 mutation. Leukemia. 2013 27(11):2247-8.
- Noris et al. Mutations in ANKRD26 are responsible for a frequent form of inherited thrombocytopenia: analysis of 78 patients from 21 families. Blood. 2011;117(24):6673-80.
- Parry et al. Syndrome complex of bone marrow failure and pulmonary fibrosis predicts germline defects in telomerase. Blood. 2011 117(21):5607-11.
- Petrovski et al. An Exome Sequencing Study to Assess the Role of Rare Genetic Variation in Pulmonary Fibrosis. Am J Respir Crit Care Med. 2017 196(1):82-93.
- Quesada et al. DDX41 mutations in myeloid neoplasms are associated with male gender, TP53 mutations and high-risk disease. Am J Hematol. 2019 94(7):757-766.
- Rio-Machin et al. The complex genetic landscape of familial MDS and AML reveals pathogenic germline variants. Nat Commun. 2020 Feb 11(1):1044.
- Savage et al. Mutations in the reverse transcriptase component of telomerase (TERT) in patients with bone marrow failure. Blood Cells Mol Dis. 2006 37(2):134-6.
- Savage et al. TINF2, a component of the shelterin telomere protection complex, is mutated in dyskeratosis congenita. Am J Hum Genet. 2008 82(2):501-9.
- Schratz et al. Somatic reversion impacts MDS/AML evolution in the short telomere syndromes bioRxiv 2021.05.26.445858.
- Sharma et al. Gain-of-function mutations in RPA1 cause a syndrome with short telomeres and somatic genetic rescue. Blood. 2022 139(7):1039-1051.
- Silhan et al. Lung transplantation in telomerase mutation carriers with pulmonary fibrosis. Eur Respir J. 2014 44(1):178-87.
- Su et al. Mutational spectrum of acute myeloid leukemia patients with double *CEBPA* mutations based on next-generation sequencing and its prognostic significance. Oncotarget. 2018 9(38):24970-24979.
- Tan et al. EFL1 mutations impair eIF6 release to cause Shwachman-Diamond syndrome. Blood. 2019 134(3):277-290.
- Tien et al. Concomitant *WT1* mutations predict poor prognosis in acute myeloid leukemia patients with double mutant *CEBPA*. Haematologica. 2018 103(11):e510-e513.
- Tsakiri et al. Adult-onset pulmonary fibrosis caused by mutations in telomerase. Proc Natl Acad Sci U S A. 2007 104(18):7552-7.
- Tummala et al. ERCC6L2 mutations link a distinct bone-marrow-failure syndrome to DNA repair and mitochondrial function. Am J Hum Genet. 2014 94(2):246-56.
- Tummala et al. Poly(A)-specific ribonuclease deficiency impacts telomere biology and causes dyskeratosis congenita. J Clin Invest. 2015 125(5):2151-60.

- Tummala et al. DNAJC21 Mutations Link a Cancer-Prone Bone Marrow Failure Syndrome to Corruption in 60S Ribosome Subunit Maturation. Am J Hum Genet. 2016 99(1):115-24.
- Tummala et al. Homozygous OB-fold variants in telomere protein TPP1 are associated with dyskeratosis congenita-like phenotypes. Blood. 2018 132(12):1349-1353.
- Tummala et al. Genome instability is a consequence of transcription deficiency in patients with bone marrow failure harboring biallelic *ERCC6L2* variants. Proc Natl Acad Sci U S A. 2018 115(30):7777-7782.
- Tummala et al. A frameshift variant in specificity protein 1 triggers superactivation of Sp1-mediated transcription in familial bone marrow failure. Proc Natl Acad Sci U S A. 2020 117(29):17151-17155.
- Tummala et al. Germline thymidylate synthase deficiency impacts nucleotide metabolism and causes dyskeratosis congenita. Am J Hum Genet. 2022 109(8):1472-1483.
- Ulirsch et al. The Genetic Landscape of Diamond-Blackfan Anemia. Am J Hum Genet. 2018 103(6):930-947.
- Vogiatzi et al. A family with Hoyeraal-Hreidarsson syndrome and four variants in two genes of the telomerase core complex. Pediatr Blood Cancer. 2013 60(6):E4-6.
- Vulliamy et al. Dyskeratosis congenita caused by a 3' deletion: germline and somatic mosaicism in a female carrier. Blood. 1999 94(4):1254-60.
- Vulliamy et al. The RNA component of telomerase is mutated in autosomal dominant dyskeratosis congenita. Nature. 2001 413(6854):432-5.
- Vulliamy et al. Association between aplastic anaemia and mutations in telomerase RNA. Lancet. 2002 359(9324):2168-70.
- Vulliamy et al. Disease anticipation is associated with progressive telomere shortening in families with dyskeratosis congenita due to mutations in TERC. Nat Genet. 2004 36(5):447-9.
- Vulliamy et al. Mutations in dyskeratosis congenita: their impact on telomere length and the diversity of clinical presentation. Blood. 2006 107(7):2680-5.
- Vulliamy et al. Mutations in the telomerase component NHP2 cause the premature ageing syndrome dyskeratosis congenita. Proc Natl Acad Sci U S A. 2008 105(23):8073-8.
- Vulliamy et al. Differences in disease severity but similar telomere lengths in genetic subgroups of patients with telomerase and shelterin mutations. PLoS One. 2011;6(9):e24383.
- Vulliamy et al. Telomere length measurement can distinguish pathogenic from non-pathogenic variants in the shelterin component, TIN2. Clin Genet. 2012 81(1):76-81.
- Walne et al. Genetic heterogeneity in autosomal recessive dyskeratosis congenita with one subtype due to mutations in the telomerase-associated protein NOP10. Hum Mol Genet. 2007 16(13):1619-29.
- Walne et al. TINF2 mutations result in very short telomeres: analysis of a large cohort of patients with dyskeratosis congenita and related bone marrow failure syndromes. Blood. 2008 112(9):3594-600.
- Walne et al. Mutations in C16orf57 and normal-length telomeres unify a subset of patients with dyskeratosis congenita, poikiloderma with neutropenia and Rothmund-Thomson syndrome. Hum Mol Genet. 2010 19(22):4453-61.

- Walne et al. Exome sequencing identifies MPL as a causative gene in familial aplastic anemia. Haematologica. 2012 97(4):524-8.
- Walne et al. Constitutional mutations in RTEL1 cause severe dyskeratosis congenita. Am J Hum Genet. 2013 92(3):448-53.
- Walne et al. Marked overlap of four genetic syndromes with dyskeratosis congenita confounds clinical diagnosis. Haematologica. 2016 101(10):1180-1189.
- Walne et al. Expanding the phenotypic and genetic spectrum of radioulnar synostosis associated hematological disease. Haematologica. 2018 103(7):e284-e287.
- Xin et al. Functional characterization of natural telomerase mutations found in patients with hematologic disorders. Blood. 2007 109(2):524-32.
- Yu et al. Identification of pathogenic germline variants in a large Chinese lung cancer cohort by clinical sequencing. Mol Oncol. 2023.
- Zhong et al. Disruption of telomerase trafficking by TCAB1 mutation causes dyskeratosis congenita. Genes Dev. 2011 25(1):11-6.
