# Supplementary figures and images for: The evolving genetic landscape of telomere biology disorder dyskeratosis congenita
Source: EMBO Mol Med. 2024 Aug 28;16(10):16. doi: 10.1038/s44321-024-00118-x (PMC11473520; doi:10.1038/s44321-024-00118-x)

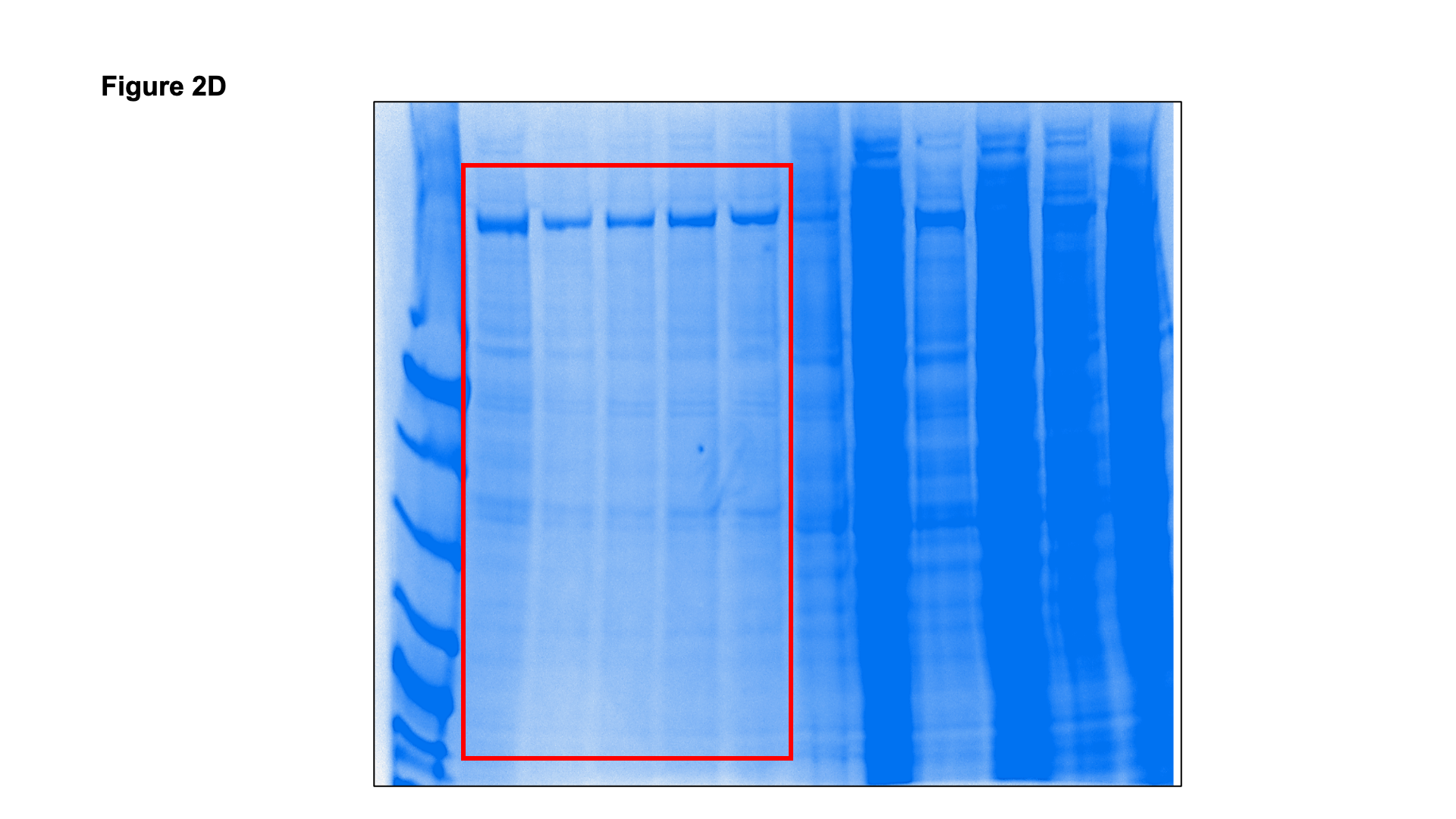

Supplement: Supplementary file 5 — Source data Fig. 2 [file 44321_2024_118_MOESM5_ESM.zip › Figure 2/Figure 2D.tiff]

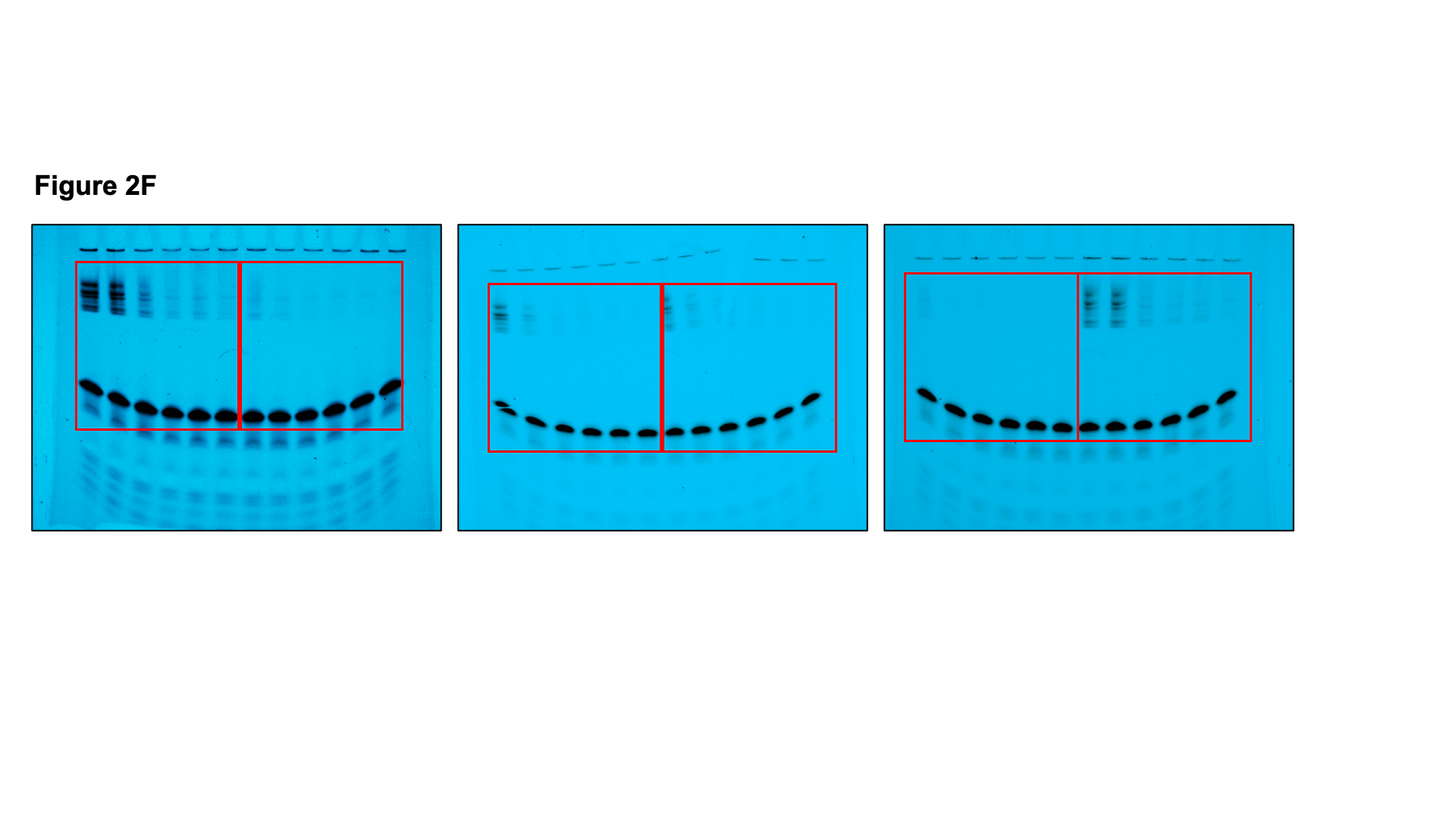

Supplement: Supplementary file 5 — Source data Fig. 2 [file 44321_2024_118_MOESM5_ESM.zip › Figure 2/Figure 2F.tiff]

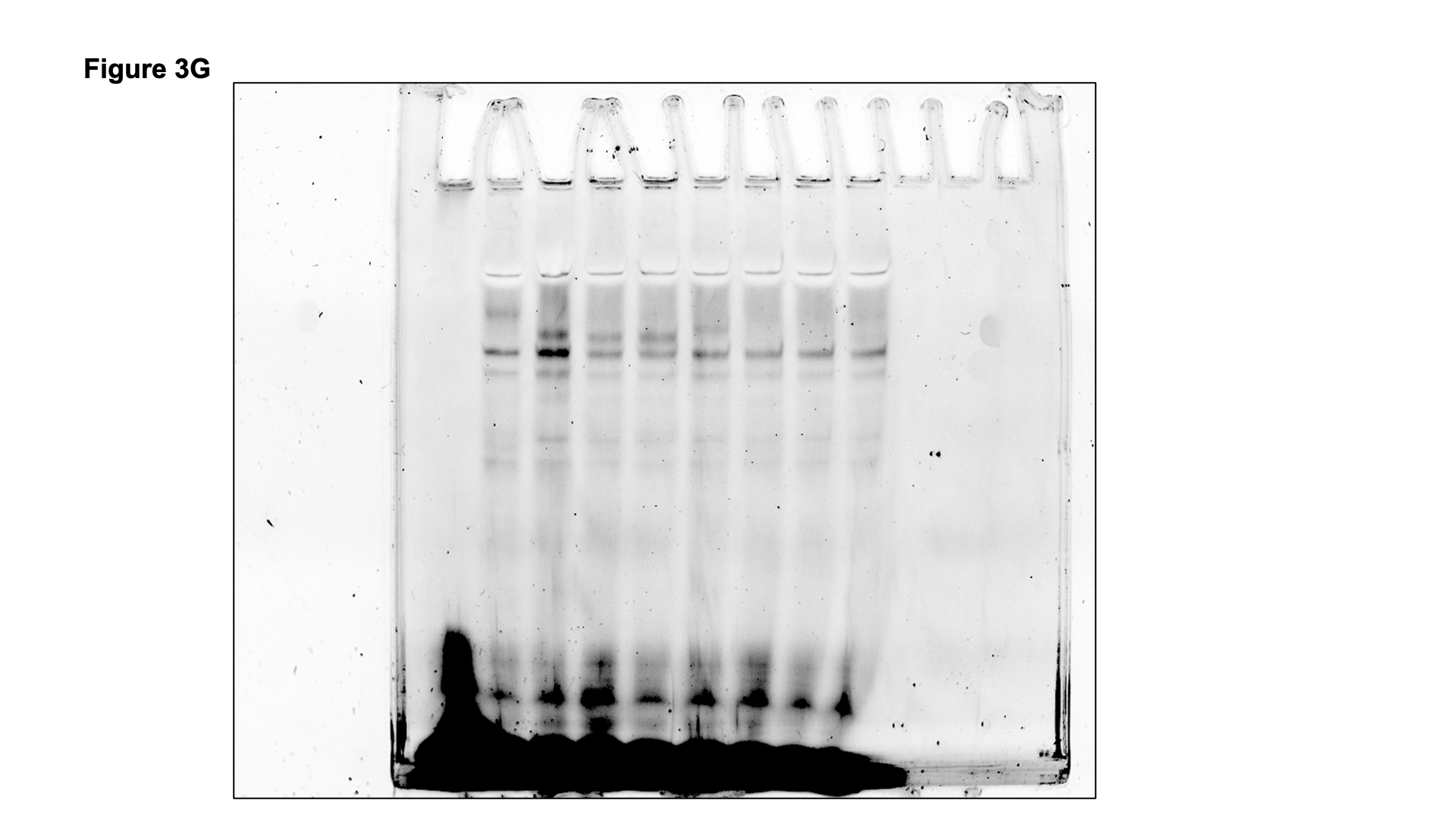

Supplement: Supplementary file 6 — Source data Fig. 3 [file 44321_2024_118_MOESM6_ESM.zip › Figure 3/Figure 3G.tiff]

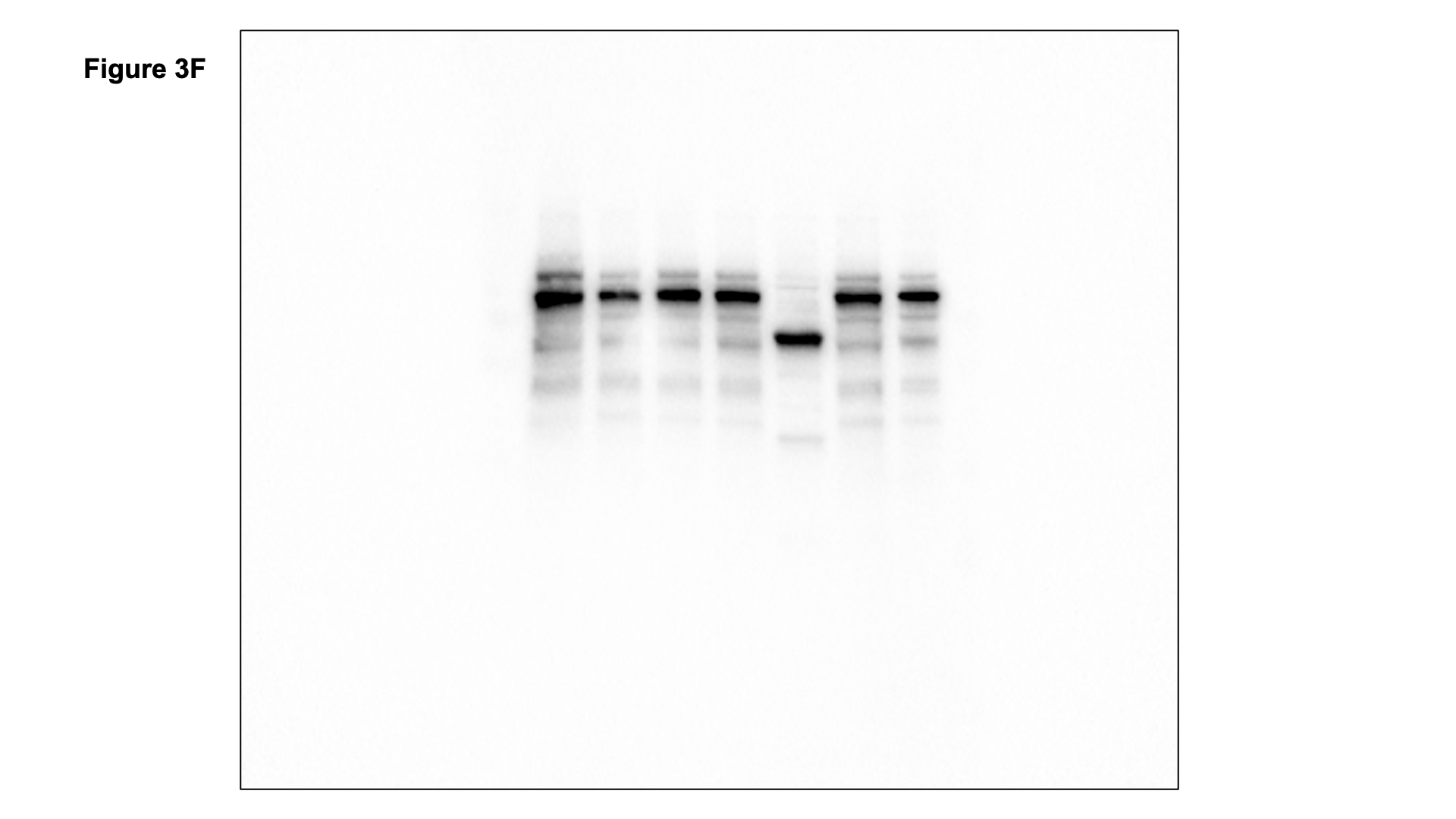

Supplement: Supplementary file 6 — Source data Fig. 3 [file 44321_2024_118_MOESM6_ESM.zip › Figure 3/Figure 3F.tiff]

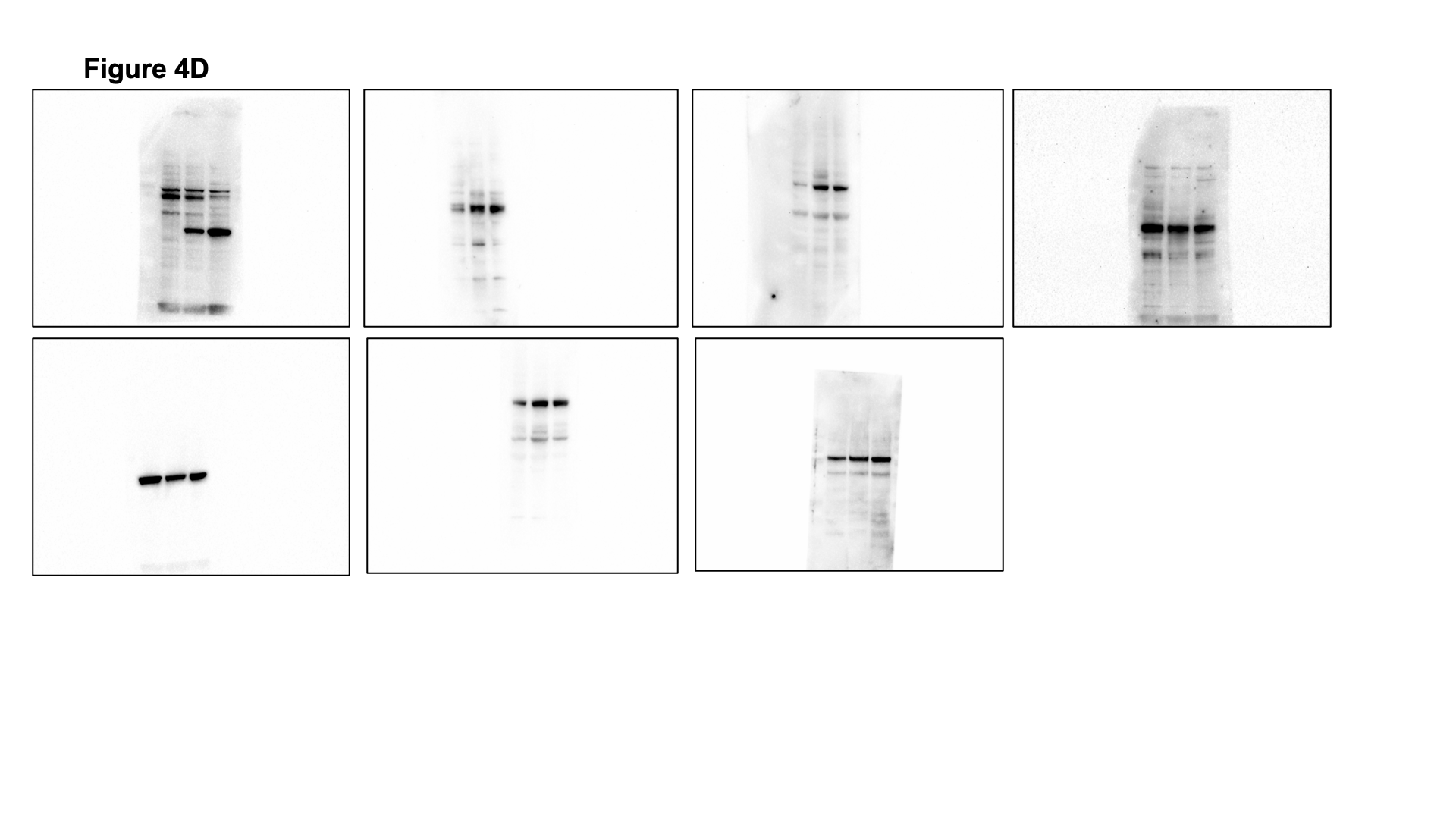

Supplement: Supplementary file 7 — Source data Fig. 4 [file 44321_2024_118_MOESM7_ESM.zip › Figure 4/Figure 4D.tiff]

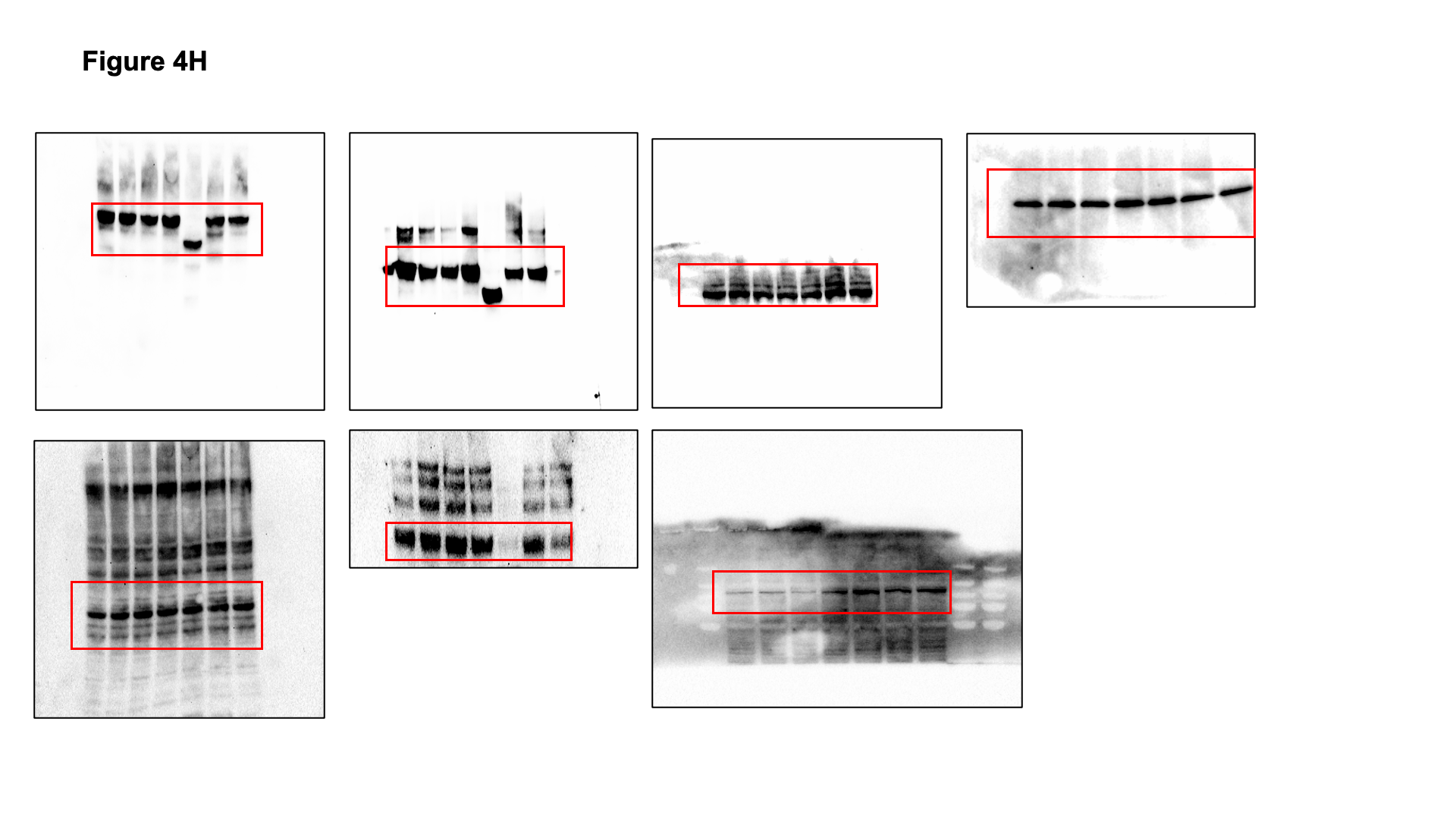

Supplement: Supplementary file 7 — Source data Fig. 4 [file 44321_2024_118_MOESM7_ESM.zip › Figure 4/Figure 4H.tiff]

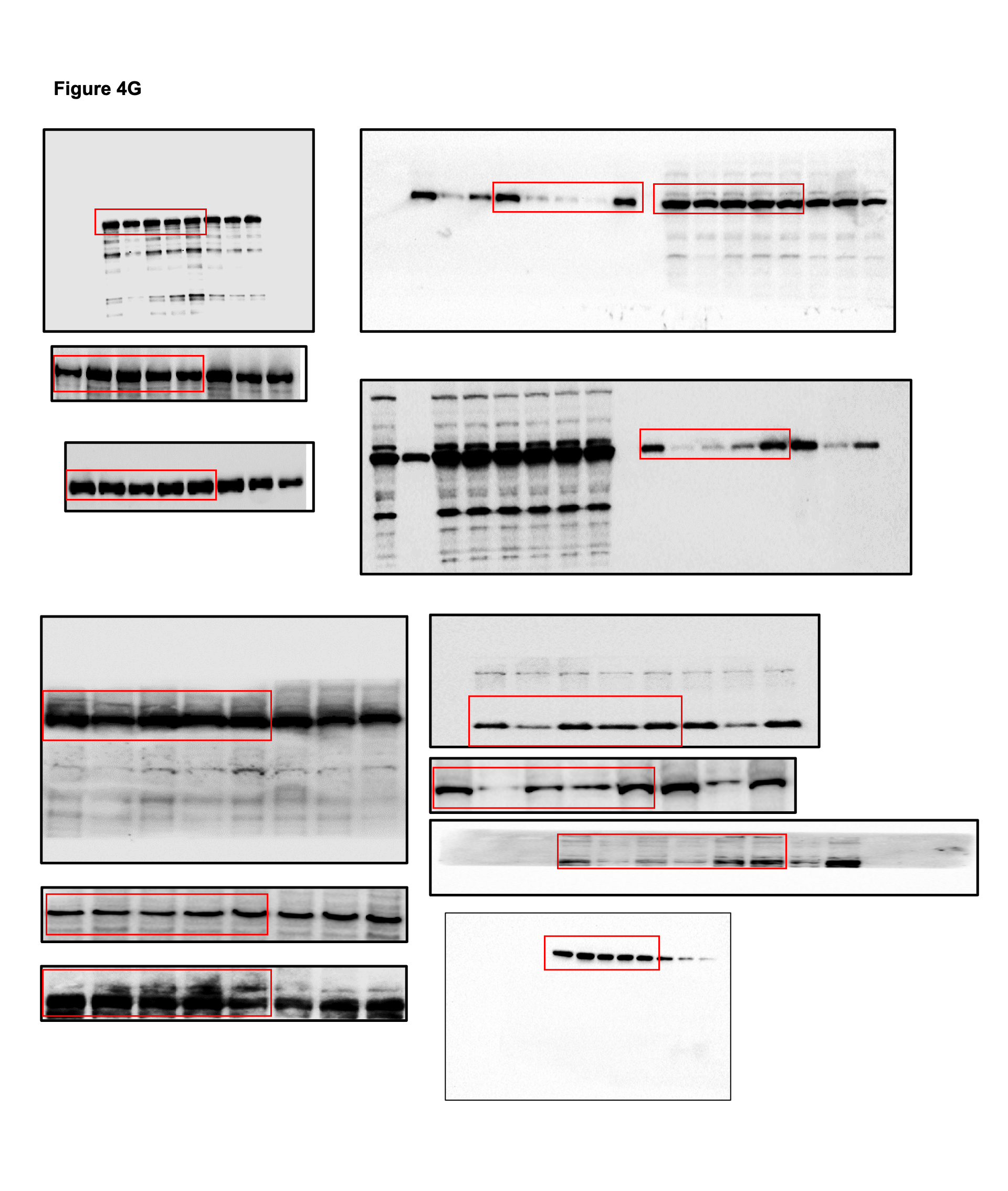

Supplement: Supplementary file 7 — Source data Fig. 4 [file 44321_2024_118_MOESM7_ESM.zip › Figure 4/Figure 4G.tiff]

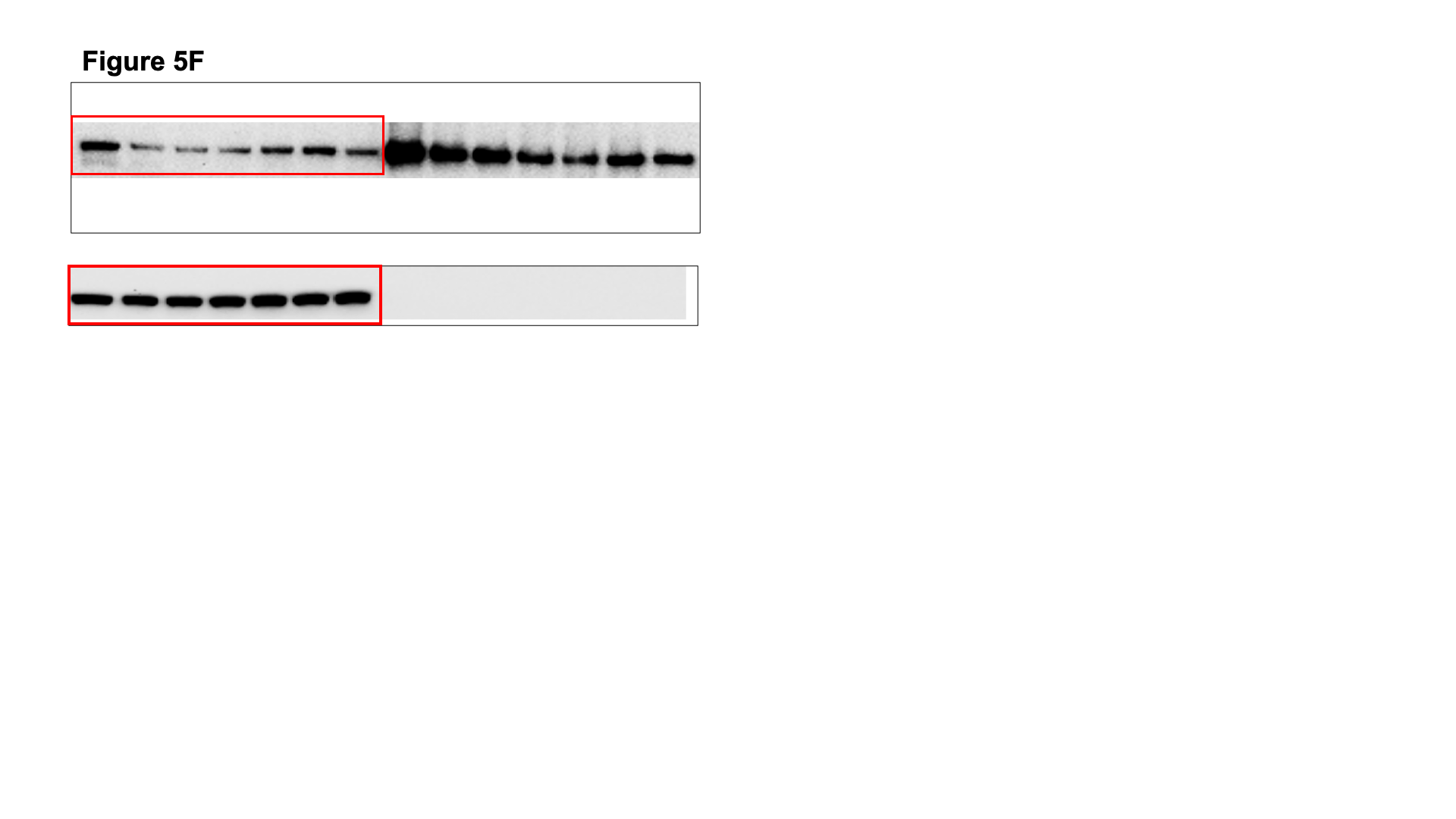

Supplement: Supplementary file 8 — Source data Fig. 5 [file 44321_2024_118_MOESM8_ESM.zip › Figure 5/Figure 5F.tiff]
