# Supplementary material for: The evolving genetic landscape of telomere biology disorder dyskeratosis congenita
Source: EMBO Mol Med. 2024 Aug 28;16(10):16. doi: 10.1038/s44321-024-00118-x (PMC11473520; doi:10.1038/s44321-024-00118-x)
Supplement: Supplementary file 11 — Expanded View Figures [file 44321_2024_118_MOESM11_ESM.pdf]

Expanded View Figures

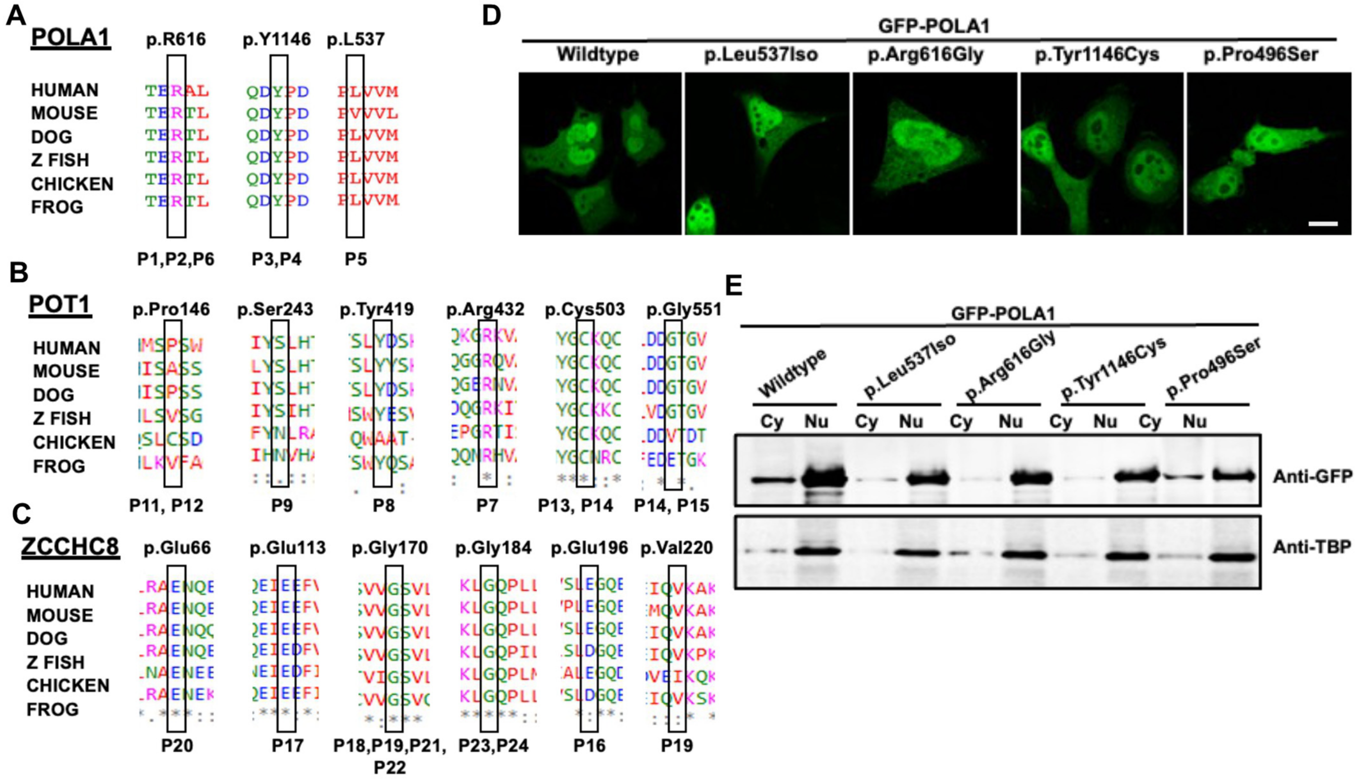

Figure EV1. Germline variants in POLA1, POT1 and ZCCHC8.

(A–C) Alignments were generated using Clustal omega, comparing the amino acid sequences around the sites of the variants identified in this study to other species. The high degree of conservation is evident, and the colour scheme indicates individual amino acid residue type assigned on basis of their profile by default parameters in Clustal omega. '*' indicates highly conserved ':' indicates semi conserved. **(D)** In HeLa cells expressing, confocal imaging of GFP-tagged POLA1 revealed predominantly nuclear and some cytoplasmic expression of POLA1. Scale bar, 20 μm. **(E)** This localisation is also confirmed by nuclear and cytoplasmic cell fractionation and subsequent western blotting. TATA-binding protein (TBP) antibody is used nuclear loading control. Source data are available online for this figure.

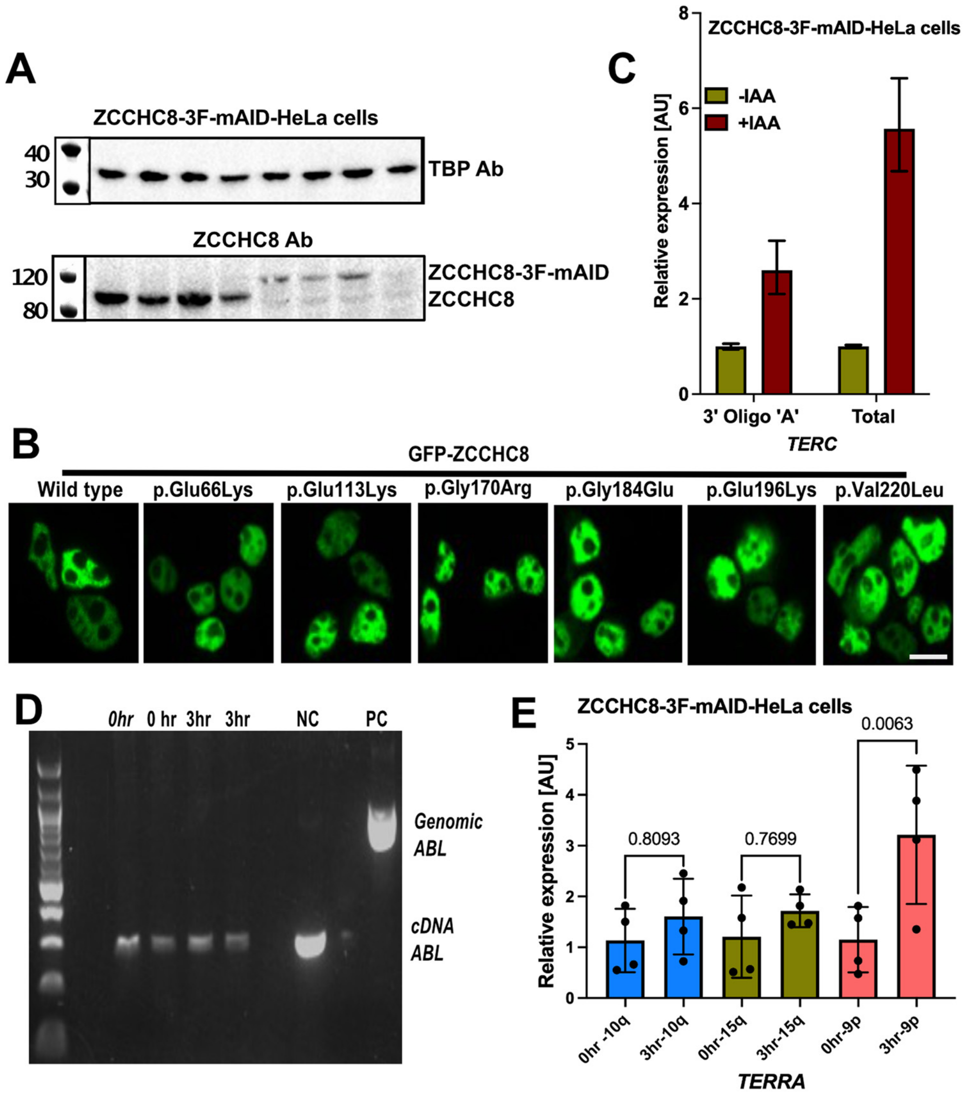

Figure EV2. ZCCHC8 depletion increases *TERC* and *TERRA* transcripts in HeLa cells.

(**A**) Immunoblot showing the reduction of ZCCHC8 protein levels after treatment with indole-3-acetic acid (IAA) at 750 μM for the indicated time points in HeLa-Tir1 cells and ZCCHC8-3F-mAID HeLa cells. TATA-binding protein (TBP) is used as a loading control. (**B**) Confocal images of GFP-tagged ZCCHC8 in HeLa cells. Panels are representative of images taken from different fields of view in three separate experiments. Scale bar, 20 μm. (**C**) Oligo-dT$_{(20)}$-primed mature (3′Oligo 'A') *TERC* RNA transcripts are distinguished from random hexamer-primed (Total) cDNA obtained from RNA samples from both untreated and treated ZCCHC8-3F-mAID HeLa cells with IAA. Data represent standard deviation calculated from means from upper and lower limits derived from $n = 2$ experiments run in triplicates for each condition. (**D**) Confirmation of RNA samples devoid of genomic DNA contamination as revealed by ABL primed transcript from cDNAs derived from untreated (0 h) and IAA treated (3 h and 24 h) ZCCHC8-3F-mAID HeLa cells. PC indicates genomic positive control. Data represent means ± standard deviation, from $n = 2$ experiments, with *P* values determined by one-way ANOVA as reported on the graph. (**E**) Telomeric repeat containing RNA transcripts (*TERRA*) transcripts at indicated chromosomal locations were detected in cDNA samples from untreated (0 h) and IAA treated ZCCHC8-3F-mAID HeLa cells. Data represent means ± standard deviation, from $n = 2$ experiments, with p values determined by one-way ANOVA as reported on the graph. Source data are available online for this figure.

 © The Author(s)

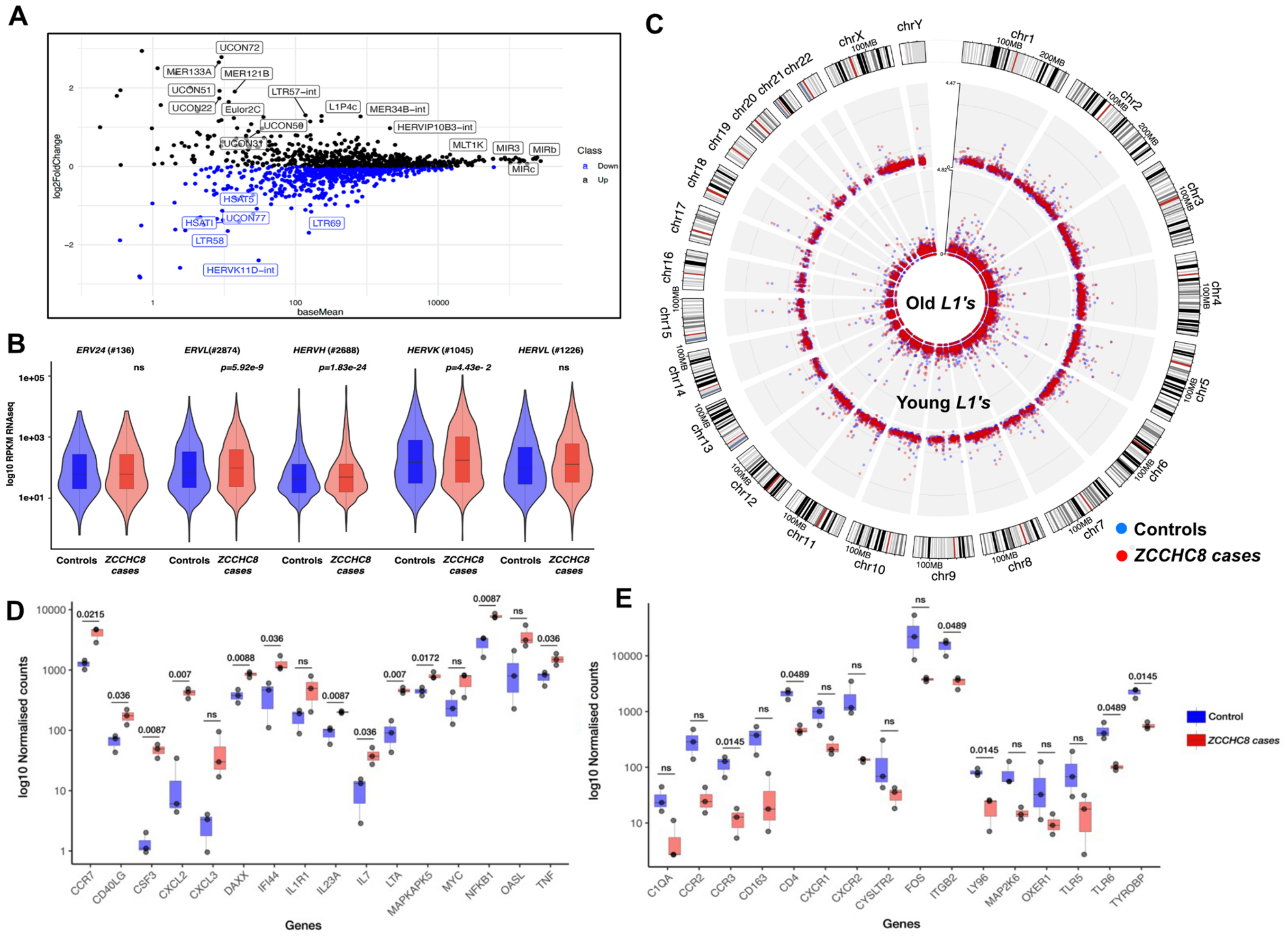

Figure EV3. Transposable elements (TE's) dysregulation in ZCCHC8 patient blood.

(**A**) Differentially expressed TE subfamilies (log2-fold change > 1 and the *P*-adjusted value < 0.05 after Benjamini–Hochberg multiple testing correction of Wald test *P* value of *ZCCHC8 cases* vs Controls; log2-fold change on y-axis and mean normalized counts on x-axis) showing either upregulated (black) or downregulated (blue). (**B**) Plots compare the expression of LTR (Long terminal repeats) subfamilies *ERV24*, *ERVL*, *HERVH*, *HERVK* and *HERVL* between Controls ($n = 3$; blue) and *ZCCHC8 cases* ($n = 3$; salmon). *P* values for all the violin/box plots were calculated using the pairwise two-sided multi-comparison Dunn test, a post hoc test, following Kruskal–Wallis test with Bonferroni correction. Violin-box plots indicate the median, bounds indicate the 25th and 75th percentiles, and whiskers limit show 1.5× interquartile range (**C**) Circos plot depicting expression of full-length L1 across chromosomal ideogram of younger L1s (*L1HS*, *L1PA2*, *L1PA3* and *L1PA4*) and older L1s (*L1PA5*-L1PA16, *L1M1-L1M4*, *L1P1-L1P4*) as RPKM levels for Controls ($n = 3$; blue) and *ZCCHC8* cases ($n = 3$; salmon). (**D**, **E**) Box plot represents differentially expressed (FDR < 0.05) inflammatory responsive genes across three independent samples of controls ($n = 3$) and *ZCCHC8* cases ($n = 3$). *X*-axis represent genes and *Y*-axis represents log10 normalised count. Violin-box plots indicate the median, bounds indicate the 25th and 75th percentiles, and whiskers limit show 1.5× interquartile range.

© The Author(s)
